# Nierenbeteiligung bei Systemerkrankungen

**DOI:** 10.1007/s00292-024-01338-1

**Published:** 2024-05-28

**Authors:** Renate Kain

**Affiliations:** Klinisches Institut für Pathologie, Medizinische Universität Wien, Währinger Gürtel 18–20, 1090 Wien, Österreich

**Keywords:** Glomerulonephritis, Vaskulitis, Systemischer Lupus erythematosus, Immun-Checkpoint-Inhibitoren, Antineutrophile Zytoplasmaantikörper, Glomerulonephritis, Vasculitis, Systemic lupus erythematosus, Immune checkpoint inhibitors, Antineutrophil cytoplasmic antibodies

## Abstract

**Hintergrund:**

Erkrankungen des nicht-neoplastischen Nierenparenchyms können im Rahmen aller, den Organismus systemisch betreffenden Erkrankungen auftreten und stellen so eine Differenzialdiagnose für immunologisch bedingte Nierenerkrankungen dar.

**Fragestellung:**

Zwei häufige autoimmunologische Erkrankungen der Niere – antineutrophile Zytoplasmaantikörper (ANCA)-assoziierte Vaskulitis (AAV) und systemischer Lupus erythematosus (SLE) – werden im Kontext des breiten Erkrankungsfelds der Nieren und deren diagnostische und mögliche therapeutische Ansätze dargestellt.

**Material und Methoden:**

Es wird eine Übersicht über die Krankheitsbilder mit Review aktueller Literatur gegeben.

## Abstract (en)

**Background:**

Diseases of the nonneoplastic renal parenchyma occur in the context of a number of disorders that affect the organism systemically and can thus represent a differential diagnosis for autoimmunological kidney diseases.

**Purpose:**

Two common autoimmunologic diseases of the kidney, namely antineutrophil cytoplasmic antibody (ANCA)-associated vasculitis (AAV) and systemic lupus erythematosus (SLE), are presented and put into context of the broader field of renal diseases. Novel diagnostic and therapeutic approaches are discussed.

**Materials and methods:**

A review of the recent literature and an overview of the disease presentation are provided.

Obwohl die Niere an einer Vielzahl (ja wenn nicht fast allen) von Systemerkrankungen beteiligt sein kann, sind es die immunlogisch bedingten Erkrankungen, in denen sie das zentral involvierte Organ darstellt. Eine potenzielle renale Beteiligung bestimmt hier die Prognose und damit den Ausgang der Grunderkrankung mit und macht neben der klinischen Abklärung gegebenenfalls eine Nierenbiopsie erforderlich. Diese oft mit einem sehr schweren Verlauf einhergehenden Erkrankungen sind Ziel intensiver Forschung und haben in den letzten Jahren nicht nur neue Biomarker aufgezeigt, sondern auch neue therapeutische Interventionen ermöglicht.

Die Niere, physiologisch angelegt als paariges Organ, ist für zentral wichtige Funktionen im Gesamtorganismus verantwortlich. Daher kann bei einer Vielzahl von Erkrankungen eine Beteiligung erwartet werden. Diese führen zu einer Dysregulation ihrer zahlreichen Aufgaben wie Ausscheidung von Stoffwechselprodukten, Regulation von Blutdruck, Blutbildung, Knochen und Mineralstoffhaushalt oder Säuren und Basenhaushalt – um nur die wichtigsten zu nennen. Darüber hinaus ist sie aufgrund der Besonderheit der feingeweblichen Strukturen und der Einzigartigkeit ihrer Zellen, wie z. B. Podozyten und Endothelzellen des glomerulären Filters, auch ein Organ, das in immunologischen Prozessen involviert ist.

## Keine systemische Erkrankung ohne Nierenbeteiligung

Generell sehen wir eine Beteiligung der Niere am häufigsten bei systemischen Erkrankungen (sowohl primär ursächlich als auch sekundär) wie Hochdruck oder Diabetes mellitus. Beide Erkrankungen und die mit ihnen assoziierten, mikro- aber auch makrovaskulär arteriosklerotischen Veränderungen sind heute Hauptursache für eine eingeschränkte Nierenfunktion und die Entwicklung einer terminalen Niereninsuffizienz. Die Prävalenz chronischer Nierenerkrankungen wird in einer Arbeit aus 2020 weltweit mit bis zu 9,8 % beziffert [10].

Das Herz-Kreislauf-System bzw. Erkrankungen von Herz und Gefäßen sind eng mit der Funktion und Pathologie der Niere verbunden. Dies ist nicht nur durch die akzelerierte Arteriolo- und Arteriosklerose bei Hochdruck mit Beteiligung der Nierenarterien bedingt, sondern auch durch eine Aktivierung des Renin-Angiotensin-Aldosteron-Systems (RAAS), der Ausschüttung von Zytokinen, wie Tumornekrosefaktor (TNF)-alpha und Interleukin (IL)6, die in der Folge zu akuter und chronischer Entzündung mit zunehmender Fibrosierung führen [27]. Bei Herzinsuffizienz tritt damit initial bzw. rezidivierend ein akutes Nierenversagen auf, das sich im Verlauf der Erkrankung in einem chronischen manifestiert. Da dieses, wie oben schon erwähnt, für einen hohen Anteil einer globalen Erkrankungslast („global disease burden“) verantwortlich ist, erfordert es therapeutische Ansätze, die Entwicklung (z. B. von Fibrose) zu verhindern oder sogar umzukehren. Entsprechend hat die Aufklärung der Mechanismen des kardiorenalen Syndroms, die Beeinflussung der Niere durch kardiovaskuläre Erkrankungen und umgekehrt, in den letzten Jahren zunehmend wissenschaftliches Interesse gefunden [29].

Zirkulatorische Ursachen (vorwiegend Hypoperfusion) werden ebenfalls für die Mehrzahl der Fälle eines akuten und chronischen Nierenversagens beim hepatorenalen Syndrom postuliert [24]. Doch auch andere metabolische Prozesse wie Adipositas werden zunehmend als Ursache renaler Erkrankungen erkannt. So ist ein Body Mass Index (BMI) > 30 signifikant mit der Entwicklung eines chronischen Nierenversagens assoziiert, das nicht nur zugrunde liegende renale Erkrankungen aggraviert, sondern charakteristischerweise eine adipositasassoziierte, fokal-segmentale Glomerulosklerose (FSGS) bedingen kann [1]. Die Pathomechanismen sind komplex, doch führt die viszerale Adipositas u. a. zu einer Kompression der Nieren, einer Aktivierung von Barorezeptoren und des RAAS, einer Erhöhung von Leptin und einer Überaktivierung des sympathischen Nervensystems [25].

Daneben ist eine Mitbeteiligung der Niere bei einer Vielzahl von malignen Erkrankungen zu nennen, allen voran jene, die mit einer Dysproteinämie mit nephrotoxischen monoklonalen Immunglobulinen (Gammopathie) assoziiert sind. Es ist hier wichtig festzuhalten, dass die diagnostischen Kriterien für ein manifestes multiples Myelom oder eine B‑Zell-Neoplasie nicht gegeben sein müssen. Allein die Beteiligung der Niere bei Auftreten einer monoklonalen Gammopathie unbestimmter Signifikanz (MGUS) definiert hier die Signifikanz der (bei weitem nicht als benigne zu bezeichnenden Erkrankung) monoklonalen Gammopathie mit renaler Signifikanz (MGRS). Sie zu erkennen erfordert in den meisten Fällen eine Nierenbiopsie, da sie ein breites Spektrum morphologischer Veränderungen umfasst, von denen exemplarisch die glomeruläre Leichtkettenspeicherkrankheit, C3- oder membranoproliferative Glomerulonephrits, eine Myelomniere oder tubuläre Kristallopathie und die Leichtkettenamyloidose zu nennen sind, die mit erhöhter Morbidität und Mortalität einhergehen [19].

Doch auch solide Tumoren und deren Therapie können die Nieren und ihre Funktion beeinflussen. So wurde gezeigt, dass z. B. bei Patient:innen mit Nierentumoren renale Erkrankungen verschiedenster Ursachen überproportional häufig sind. Allen voran sieht man renovaskuläre Erkrankungen, wie die oben genannten Veränderungen bei Diabestes mellitus und Hypertension, aber auch Glomerulonephritiden und tubulointerstitielle Nephritiden. Entsprechend ist eine Untersuchung des nicht-neoplastischen Nierenparenchyms (idealerweise zumindest 1 cm vom Tumorrand entfernt) empfohlen, da die dort festgestellten Erkrankungen die weitere Funktion des verbleibenden Parenchyms oder der Niere entscheidend beeinflussen [13, 18].

Auch moderne, personalisierte Therapien maligner Erkrankungen, wie die Immun-Checkpoint-Inhibitoren (ICI), monoklonale Antikörper, die gegen inhibitorische Rezeptoren auf Leukozyten gerichtet sind, können immunologische Phänomene auslösen. Diese können zu einer Nierenerkrankung (wie interstitielle Nephritis oder verschiedene Formen einer Glomerulonephritis, GN) führen [26].

Die genannten Erkrankungen sind hier nicht primärer Inhalt, sie müssen jedoch immer in der Bewertung von Nierenbiopsien miteinbezogen werden, da sie (wie Hochdruck, Diabetes mellitus oder Adipositas) häufig sind und differentialdiagnostisch als Ursache für genetische oder (auto)immunologisch bedingte renale Erkrankungen in Betracht gezogen werden müssen.

Auf die Niere selbst als Angriffspunkt eines immunologischen Geschehens wird im Folgenden im Hinblick auf neue Entwicklungen in Pathogenese, Diagnostik und Therapie näher eingegangen. Obwohl die Niere im Prinzip bei sämtlichen immunologischen Erkrankungen involviert sein kann (beispielhaft seien hier z. B. Sarkoidose oder IgG 4-mediierte Erkrankungen genannt), sind es v. a. zwei Erkrankungen, die klassischerweise als immunologische Systemerkrankungen gesehen werden. Dies sind die systemischen Vaskulitiden kleiner Gefäße und die prototypische Autoimmunerkrankung systemischer Lupus erythematosus (SLE). Obwohl man meist primär an glomeruläre Manifestationen in der Niere denkt, betreffen beide Erkrankungen sämtliche Kompartimente der Niere, also auch den tubulointerstitiellen Raum und natürlich die Gefäße, deren hoch spezialisierte Abschnitte die Glomeruli darstellen.

## Vaskulitis kleiner Gefäße – antineutrophile Zytoplasmaantikörper (ANCA)-assoziierte Vaskulitis (AAV)

Entsprechend der 1994 erstmals erstellten und 2012 revidierten „Chapel Hill Consensus Conference (CHCC) Nomenklatur“ umfassen die Vaskulitiden kleiner Gefäße ein breites Spektrum von Erkrankungen, bei denen vorwiegend die kleinsten Gefäße (also die Kapillaren) betroffen sind, jedoch auch größere Gefäße betroffen sein können [16]. Es handelt sich dabei nicht um eine Klassifikation oder diagnostische Kriterien, sondern sie diente dem Zweck, eine Nosologie für Beschreibungen und Definitionen einzelner Erkrankungen zu erstellen, um Patient:innen für Studien zu stratifizieren. Dies gilt ebenfalls für die 1990 publizierten Kriterien zur Klassifikation des American College of Rheumatology [15]. Entsprechend den Ergebnissen wissenschaftlicher Studien werden diese Kriterien laufend einer Evaluierung unterzogen, um einer ätiologischen Klassifikation näher zu kommen. Grundlagenforschung und klinischen Studien ist es auch zu verdanken, dass in den letzten Jahren verbesserte Behandlungsregimes und Therapieansätze entwickelt werden konnten [17].

Klinisch gehören dazu im engeren Sinn die mikroskopische Polyangiitis, die Granulomatose mit Polyangiitis bzw. die eosinophile Granulomatose mit Polyangiitis. Insgesamt findet man aber auch Erkrankungsbilder wie Antibasalmembran-GN, hypokomplementämische Vaskulitis und IgA-Vaskulitis abgebildet, auf die jedoch nicht weiter eingegangen wird. Diese Gruppe seltener Erkrankungen ist meist mit Antikörpern gegen Myeloperoxidase und Proteinase 3 assoziiert, den klassischen Antigenen der ANCA. Die serologische ANCA-Diagnostik beruht einerseits auf einer indirekten Immunfluoreszenz mit Patient:innensera auf Granulozyten gesunder Spender und antigenspezifischen ELISA („enzyme linked immunosorbent assay“), wobei letztgenannte Tests heute primär als Standard eingesetzt werden sollen [21].

**Abb. 1:**
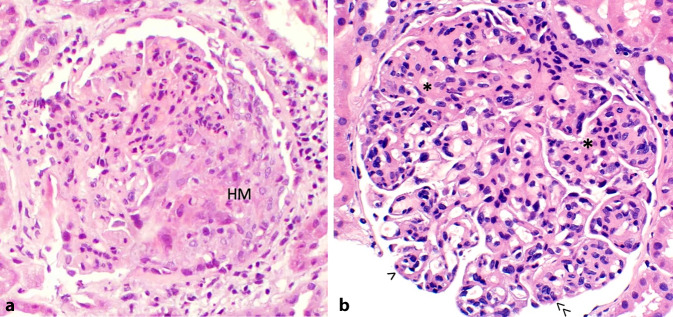
Glomeruläre Veränderungen bei fokal nekrotisierender Glomerulonephritis (FNGN; **a**) oder Lupusnephritis (**b**). Bei FNGN bestehen halbmondartige Proliferationen der Epithelzellen (*HM*), sehr kleine Nekrosen können in der H&E-Färbung (Hämatoxilin-Eosin) manchmal schwer detektierbar sein, zur besseren Darstellung kann eine Trichromfärbung hilfreich sein. Für Lupusnephritis sind mesangiale Expansion und Zellproliferation (*Asterisk*), endokapilläre Proliferation (≫) und verbreiterte Kapillarschlingen (>) charakteristisch (H&E, Vergr. 1:40)

In diesen Erkrankungen ist nicht nur die Niere eines der Hauptzielorgane der immunologisch bedingten Schädigung, sondern ihre Involvierung definiert Organüberleben und Mortalität insgesamt. Bei unklarem Krankheitsbild oder um das Ausmaß renaler Involvierung und Schädigung zu evaluieren, ist nach wie vor die histopathologische Biopsiediagnostik Standard. In der Niere ist die definierende Veränderung die nekrotisierende und halbmondbildende („crescentic“) GN (Abb. 1a). Deren früheste Veränderungen gehen mit segmentaler Nekrose glomerulärer Kapillarschlingen und Exsudation von Fibrin und Blutbestandteilen einher.

**Abb. 2:**
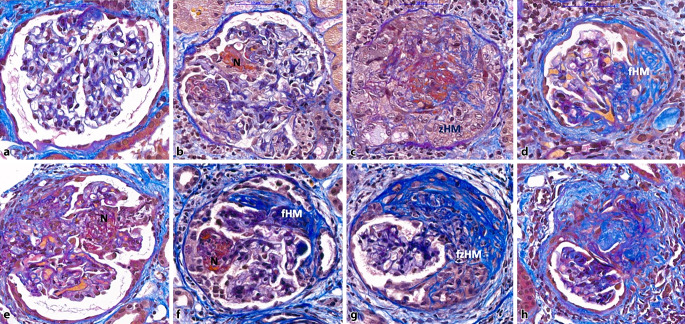
Die Evolution glomerulärer Läsionen bei antineutrophiler Zytoplasmaantikörper (ANCA)-assoziierter Vaskulitis (AAV): Neben weitgehend unauffällig erscheinenden Glomeruli (**a**) können Nekrosen (*N*) einzelner Schlingen (**b**) oder ausgedehnte nekrotisierende Veränderungen mit zellulären Halbmonden (*zHM*) zu sehen sein (**c**). In der Folge fibrosieren die Halbmonde (*fHM*) unter Beteiligung der Kapillarschlingen (**d**), wobei wiederum frische Nekrosen mit neuerlicher Epithelproliferation und Halbmondbildung auftreten (**e–g**). Bei > 50 % Sklerosierung der glomerulären Segmente liegt eine globale Schwielenbildung vor (**h**). Sind in einer Biopsie > 50 % normale Glomeruli (**a**) enthalten, wird sie entsprechend der Klassifikation nach Berden et al. [5] der fokalen Kategorie, bei > 50 % Glomeruli mit zellulären Halbmonden (**c,** **d**) der Halbmondkategorie („crescentic“) und bei > 50 % Glomeruli mit > 80 % verschwielten Schlingen (**g,** **h**) der sklerosierenden Gruppe zugeordnet. Findet man keines der vorherrschenden Bilder, werden die Veränderungen der gemischten Gruppe zugeordnet (Saures Fuchsin-Orange G [SGOG], Vergr. x40; *fzHM* fibrozellulärer Halbmond)

Zu Beginn der Erkrankung sind oft nur einzelne Glomeruli betroffen, was in der Bezeichnung fokal nekrotisierende GN (FNGN) ihren Ausdruck findet. Gefolgt von einer Proliferation der glomerulären Epithelzellelemente – die einen sog. Halbmond bilden – mit leukozytärer Infiltration und nachfolgender Schlingensklerose, führt der meist rezidivierende Verlauf mit immer wieder frischen Nekrosen und Halbmonden zu einer zunehmenden Vernarbung der Glomeruli und der zugehörigen Tubulusabschnitte ([2]; Abb. 2). Die Evolution dieser Veränderungen ist in der von Berg et al. [5] entwickelten Klassifikation reflektiert. Eine Beteiligung von bis mitttelgroßen intrarenalen Arterien ist nur in einem geringen Teil der Biopsien vorhanden, wenn sie jedoch zu sehen sind, so meist mit segmentaler Nekrose aller Wandschichten, selten mit granulomatöser Entzündung.

## Prädiktive Diagnostik bei AAV

Allen Krankheitsbildern gemeinsam ist, dass zu Beginn der Erkrankung der Verlauf nicht vorhersehbar und die therapeutische Beeinflussbarkeit (v. a. mit Immunsuppressiva wie Glukokortikoiden und Cyclophosphamid) unsicher ist. Ultimativ ist jedoch das Ziel der Präzisionsmedizin eine maßgeschneiderte Therapie der Erkrankung bei gleichzeitiger Minimierung der Nebenwirkungen. Auch wenn die klinische Präsentation vorranging für die Diagnose ist und der Nachweis von ANCA diese unterstützt, ist die Suche nach prognostischen Biomarkern, die eine Krankheitsaktivität anzeigen sollen, laufend Gegenstand wissenschaftlicher Arbeiten. So wurde rezent der Nachweis von CD163 im Urin allein oder in Kombination mit dem „monocyte chemoattractant protein-1“ [22] als Marker für eine aktive Erkrankung beschrieben, da ANCA-Titer hier eine schlechte Korrelation zeigen.

Auch auf Basis histomorphologischer Veränderungen wurden Klassifizierungsschemata entwickelt, die prädiktive Aussagen über den Krankheitsverlauf ermöglichen sollen. So wurden 2010 von Bergen et al. [5] aus Leiden Klassifikationskriterien entwickelt, die entsprechend der Schwere bzw. Art der Veränderungen in Nierenbiopsien vier Klassen der Erkrankung differenziert haben (Abb. 2). Ein weiterer, von Brix et al. [7] entwickelter, renaler Risikoscore bezieht nicht nur die Anzahl normaler Glomeruli, sondern auch die interstitielle Fibrose und tubuläre Atrophie sowie als klinischen Parameter die eGFR („estimated glomerular filtration rate“) zum Zeitpunkt der Diagnose ein. Die Patient:innen werden dabei in Gruppen mit einem niedrigen, mittleren oder hohen Risiko, innerhalb von 36 Monaten ein terminales Nierenversagen zu entwickeln, unterteilt.

In einer Arbeit der französischen Vaskulitisstudiengruppe [6] wurde die Histopathologie und Verlauf bei 251 Patient:innen evaluiert und der Frage nachgegangen, ob entweder keine oder aktive Veränderungen im Sinne von Nekrosen und Entzündung größerer Gefäße in der Niere bei AAV relevant sind. So konnte gezeigt werden, dass Patient:innen mit aktiver Arteriitis älter waren, eine stärkere inflammatorische Antwort zeigten, oft eine periphere Neuropathie und gastrointestinale Symptome hatten. Es bestand keine Assoziation mit ANCA-Serotyp, eGFR, der Proteinausscheidung oder einer Dialyseabhängigkeit. Bei einer Verlaufskontrolle nach 3 Jahren war das Vorhandensein einer Arteriitis jedoch mit einem schlechteren, terminale niereninsuffizienzfreien Überleben assoziiert. Dies könnte darauf hinweisen, dass der Befall einer Nierenarterie eine generalisierte Vaskulitis indiziert.

Zunehmend wird auch die chronische Schädigung der Niere als Parameter für das Überleben des Organs herangezogen. So gewinnt die Einbeziehung einer prozentuellen bzw. semiquantitativ evaluierten Fibrosierung aller Kompartimente, wie Glomerulosklerose, interstitieller Fibrose mit tubulärer Atrophie und Arteriosklerose an Bedeutung. Darauf basiert ein weiteres histologisches Graduierungssystem, der sog. Mayo Clinic Chronicity Score (MCCS; [9]), der jedoch auch für Biopsien aus einem breiten Spektrum von Erkrankungen umgesetzt werden kann.

Auf renale Veränderungen bei AAV angewandt, konnte in einer Arbeit aus der Mayo Clinic gezeigt werden, dass der summarische Score aus allen Veränderungen mit der eGFR als Marker für den Grad des Nierenversagens zum Zeitpunkt der Diagnose korreliert. So erlaubte der Score auch eine Stratifizierung der Patient:innen hinsichtlich des Verlaufs, dem Überleben des Organs und dem Zeitraum bis zum terminalen Nierenversagen oder Tod.

Die Wertigkeit wiederholter Biopsien wurde von Hruskova et al. [14] in einer retrospektiven Studie unter Einschluss von 57 Patient:innen mit AAV untersucht. So zeigten Zweitbiopsien (protokollmäßig im Schnitt 130 Tage nach der Erstbiopsie durchgeführt) wenig überraschend, dass nach Einsetzen der Induktionstherapie in der Zweitbiopsie weniger aktive und mehr chronische glomeruläre und tubulointerstitielle Veränderungen gefunden wurden. Bekannte Biomarker wie Hämaturie korrelierten nicht mit der Aktivität in den Nierenbiopsien. Sowohl die Scores der Berden- als auch der Brix-Klassifikation änderten sich in 50 % der Patienten zwischen Erst- und Zweitbiopsie. Als Instrument der Vorhersage der Entwicklung eines terminalen Nierenversagens betrachtet, waren die Scores in der Zweitbiopsie besser prädiktiv, v. a. jene mit vorwiegend sklerotischen Veränderungen oder einem hohen Risikoindex.

Insgesamt zeigen alle Studien und Publikationen zu histopathologischen Veränderungen, dass sämtliche Kompartimente der Niere betroffen sind und entsprechend beurteilt werden müssen. Während Klassifizierungsschemata bei Lupusnephritis, wie unten ausgeführt, bereits lange etabliert sind und einige der genannten Klassifizierungen oder Scores in einer kleinen Anzahl von Studien bei AAV reproduziert wurden, sind die Ergebnisse teils widersprüchlich und es bedarf weiterer Studien zu deren Validierung und klinischen Testung. Auch muss die Wertigkeit rein morphologisch beurteilter Veränderungen immer im Kontext der Biologie der Erkrankung gesehen werden. Hier haben rezent entwickelte Methoden, wie In-situ-Transkriptomik oder Proteomik („spatial profiling“), vielversprechende Ergebnisse gezeigt [23] und die Anwendung künstlicher Intelligenz in der Evaluierung von Nierenbiopsien lässt hier eine interessante Entwicklung erwarten.

## Systemischer Lupus erythematosus (SLE) – Lupusnephritis

Die Lupusnephritis ist die glomeruläre Manifestation einer komplexen chronischen Autoimmunerkrankung, die prinzipiell jedes Organ betreffen kann, bei der mit 60 % die Niere das am häufigsten betroffene Organ ist. Die renale Manifestation bei SLE bestimmt auch hier den Verlauf bzw. Morbidität und Mortalität. Die Erkrankung betrifft vorwiegend jüngere Frauen, kann aber in allen Altersgruppen gesehen werden. Definiert bzw. charakterisiert wird sie durch Antikörper gegen nukleäre Komponenten inklusive Anti-DNS-Antikörper aber auch Antikörper gegen Komplementbestandteile wie C1q. Therapeutische Regimes wie Immunosuppression unter Gabe von Cyclophosphamid und Mycophenolat-Mofetil sowie der Einsatz von Biologika haben die Mortalitätsrate in den letzten 30 Jahren deutlich gesenkt.

Histologisch ist die Lupusnephritis durch das Fehlen einheitlicher morphologischer Veränderungen gekennzeichnet. Entsprechend wird sie auch als „Harlekin“ der glomerulären Erkrankungen betrachtet, da alle Reaktionsmuster und Formen einer GN gesehen werden können (Abb. 1b). Gemeinsam ist jedoch allen Manifestationen das sog. Full-house-Muster, also eine Ablagerung von Immunkomplexen, die routinemäßig diagnostisch detektiert werden und alle Klassen von Immunglobulinen (vorwiegend, IgG, IgM und IgA) und Komplementfaktoren C3c und C1q enthalten. Hierbei ist gerade die deutlich nachweisbare Positivität von C1q für die Lupusnephritis charakteristisch. Man unterscheidet entsprechend einer breit angewandten und laufend (zuletzt 2018) revidierten Klassifikation [4], 6 histologische Klassen von I bis VI entsprechend den Empfehlungen der International Society of Nephrology/Renal Pathology Society. Diese Klassifikation hat im Laufe der Jahre ihren Wert als richtungsweisend für die Therapie und auch für die Entwicklung klinischer Studien bewiesen und ist derzeit von Bajema et al. (persönliche Kommunikation) in geplanter Revision.

Prinzipiell basiert die Zuordnung zu den Klassen der Lupusnephritis auf dem Ausmaß der mesangialen und endokapillären Veränderungen und bezieht graduell die Vermehrung der Matrix bzw. Sklerosierung und das Ausmaß der Zellproliferation in die Evaluierung ein. Die zuletzt erstellte Klassifikation enthält neue und klarer definierte Kriterien für die mesangiale oder endokapilläre Sklerose und die Proliferation und Definition von Halbmonden, die zuvor oft unterschiedlich interpretiert wurden und zur Zuordnung zu unterschiedlichen Klassen durch Patholog:innen geführt haben. Entsprechend dem Gewicht der Zellproliferation bzw. der Sklerosierung unterscheidet man aktive und chronische Läsionen. Diese haben, unter Berücksichtigung des Umstands, dass nicht nur ein Kompartiment der Niere in einer systemischen Erkrankung betroffen ist, zur Etablierung eines bereits 1983 von Austin et al. [3] postulieren Aktivitäts- und Chronizitätsindex geführt, der die aktiven und chronischen glomerulären, tubulointerstitiellen und vaskulären Läsionen evaluiert.

Dieser Index wurde ebenfalls in der neuen Klassifikation revidiert, da er auch immer wieder in Frage gestellt wurde. So konnte in einer Studie mit 28 Patienten mit proliferativer Lupusnephritis gezeigt werden, dass bei einem Drittel der Patient:innen, die eine Rebiopsie hatten, trotz klinischer Verbesserung noch immer ein hoher histologischer Aktivitätsscore vorlag. Zwei Drittel der Patient:innen in histologischer Remission waren jedoch noch immer klinisch aktiv [20]. Entsprechend ist auch bei der Lupusnephritis die Suche nach Biomarkern, wie andere (pathogene) Antikörper, im Zentrum intensiver Untersuchungen. So ist die Relevanz gleichzeitig bestehender ANCA unklar, da bei Patient:innen mit einer Klasse-IV-Lupusnephritis und ANCA-Positivität (in > 82 % zumeist Anti-Myeloperoxidase [MPO]-Antikörper) zwar vermehrt Nekrosen und Halbmonde vorliegen und sie klinisch einen schwereren Verlauf zeigen, dies aber trotz Gabe von Cyclophosphamid keinen Einfluss auf den Ausgang der Erkrankung hatte. Daraus kann also lediglich geschlossen werden, dass ANCA für die Ausprägung der glomerulären Pathologie verantwortlich sind [30].

## Neue therapeutische Ansätze

Wie oben ausgeführt, ist die Therapie einer AAV und der Lupusnephritis von der Unterdrückung des Immunsystems, Eliminierung pathogener Antikörper und Verhinderung von deren Produktion geprägt, wobei eine Induktions- von der Erhaltungstherapie abgelöst wird, um einen Relaps zu verhindern. Im Einsatz sind Therapieschemata mit „klassischen“ Immunsuppressiva wie Glukokortikoide, Cyclophosphamid, Methotrexat oder Mycophenonolat-Mofetil, doch gerade die Langzeittherapie geht mit einer hohen Morbidität und Mortalität einher [17]. Dies erfordert neue Behandlungsansätze, unter denen sich z. B. Therapeutika aus der Onkologie, wie Rituximab in der Induktionstherapie, erfolgreich gezeigt haben. Ein anderer, vielversprechender Ansatz resultiert aus neuen Erkenntnissen zur Funktion und Pathologie des Komplementsystems.

## Komplementmediierte glomeruläre Erkrankungen

Das Komplementsystem ist in vielen, wenn nicht in den meisten Erkrankungen der Niere aktiviert und spielt einerseits eine pathogenetische, anderseits eine erkrankungsmodulierende Rolle. Primär in die Pathogenese involviert und ätiologisch gesichert ist eine Aktivierung des Komplementsystems bei den komplementassoziierten Erkrankungen im eigentlichen Sinn, tubulointerstitiellen Erkrankungen und der (antikörpermediierten) Transplantatabstoßung. Die klassischen komplementmediierten Erkrankungen (meist durch genetische Variationen in einzelnen Komplementfaktoren oder durch eine Kombination von genetischen Varianten bedingt) sind C3-GN und das atypische hämolytisch-urämische Syndrom (aHUS; [11]). Diese Erkrankungen sind extrem selten und bedingt durch eine Dysregulation bzw. Überaktivierung der Aktivierungswege des Komplementsystems.

Die C3-GN zeigt (ähnlich wie die Lupusnephritis) unterschiedliche histomorphologische Reaktionsmuster, denen jedoch allen eine prominente Deposition des Komplementfragments C3 ohne Immunglobulindeposition gemeinsam ist. In älteren Patient:innen ist die C3-GN oft mit einer monoklonalen Gammopathie assoziiert. Beim atypischen hämolytisch-urämischen Syndrom kommt es zu einer Schädigung der Endothelzellen mit einer überschießenden Thrombenbildung in kleinen Gefäßen, die oft mit einer Verbrauchskoagulopathie einhergeht. Übergänge von C3-GN zu aHUS und umgekehrt wurden jedoch berichtet. Bis heute ist nicht ganz klar, welche genetischen Faktoren für die eine oder andere Krankheit prädisponieren.

Neben einer Reihe von genetischen Defekten, die den alternativen Komplementweg aktivieren, sind die erworbenen Defekte des Komplementsystems (z. B. im Rahmen von Infektionen) in der Mehrzahl. Die Pathogenese ist dabei komplex und eine Ätiologie lässt sich nicht immer feststellen. Oft sind es jedoch Kombinationen genetischer und erworbener Faktoren, wie Infektionen, die zu einer Dysregulation des Komplementsystems führen und auch die klinische Variabilität in der Ausprägung erklären.

Unabhängig von der Erkrankung sind die molekularen Trigger der Komplementaktivierung und ihre nachfolgenden Mechanismen der Nierenschädigung unterschiedlich. So sind alle drei Aktivierungswege des Komplementsystems (der klassische, der Lektin- und der alternative Weg) und der Komplementkaskade im Rahmen von Nierenerkrankungen möglich. Vereinfacht sind es beim klassischen Aktivierungsweg Immunglobuline oder Immunkomplexe, die das Komplementsystem aktivieren. Beim Lektinaktivierungsweg sind es Proteine wie das mannosebindende Lektin, das an spezifische Zuckermoleküle bindet, während beim alternativen Aktvierungsweg eine Konvergenz der anderen zwei Wege stattfindet und diese amplifiziert. Entsprechend können auch therapeutische Ansätze an unterschiedlichen Punkten der Kaskade angreifen.

Eine Aktivierung des Komplementsystems wird bei einer Vielzahl von Erkrankungen gesehen, wie z. B. einer immunkomplexmediierten GN, der ANCA-assoziierten Vaskulitis und IgA-Nephritis, aber auch Systemerkrankungen wie die Lupusnephritis. Neben der Bildung von Immunkomplexen können Komplementbestandteile auch an die Oberfläche von Nierenzellen binden und führen dort zu einer Schädigung der Nierenzellen. So führt z. B. die Spaltung des Komplementfaktors C5 zur Bildung von C5b, dem initialen Molekül eines aus mehreren Komplementfaktoren bestehenden, sog. „membrane attack complex“ (MAC; C5b-9). Dieser bildet Poren in die Zellmembran und führt damit zum Zelltod.

## Komplementinhibition

Eine Vielzahl der heute im Einsatz befindlichen Inhibitoren des Komplementsystems stammen aus der Forschung und dem Einsatz bei den klassischen komplementassoziierten Erkrankungen [11, 17]. Der Rolle des Komplementsystems in der Pathogenese und für eine mögliche therapeutische Intervention bei anderen renalen bzw. glomerulären Erkrankungen wurde jedoch lange zu wenig Aufmerksamkeit gewidmet. Dies beruhte, wie bei FNGN, auf einer Unterschätzung oder Fehlinterpretation durch z. B. Verwendung des Begriffs „pauciimmun“, der die oft fehlenden oder in nur geringen Mengen nachweisbaren Ablagerungen von Immunkomplexen in den Glomerula von Nierenbiopsien beschreibt [12].

Basierend auf der Arbeit von Xiao et al. [31], die wegweisend die Rolle des Komplementsystems in der Pathogenese der FNGN aufzeigte, wurde in der Folge der Inhibitor des Komplementrezeptors für C5a (C5aR) für die Therapie dieser Erkrankung evaluiert. Weitere Ergebnisse aus der Grundlagenforschung und klinischer Studien führten innerhalb von 14 Jahren zu einer Zulassung des C5aR-Antagonisten Avacopan bei FNGN [14]. Sein Einsatz für die IgA-Nephritis wird zurzeit getestet [8, 28].

Der Einsatz von Komplementinhibitoren bei Lupusnephritis ist derzeit noch auf Fälle mit assoziierter thrombotischer Mikroangiopathie beschränkt bzw. wird in klinischen Phase-II-Studien getestet [28]. Damit ist der Einsatz dieser Gruppe von Therapeutika bei glomerulären Erkrankungen als Erfolg der Übersetzung von Ergebnissen aus der Grundlagenforschung über klinische Studien in den Einsatz am Patienten zu werten.

## Fazit für die Praxis

- Die Niere als zentrales Organ multipler Funktionen im Organismus ist praktisch in allen, diesen systemisch betreffenden Erkrankungen in das Krankheitsgeschehen involviert.
- Da dies auch maligne Erkrankungen betrifft, ist es wesentlich, z. B. bei Nierentumoren nicht-neoplastisches Parenchym zu untersuchen und zu befunden.
- Die Mitbeteiligung der Niere im Rahmen verschiedenster Erkrankungen stellt immer eine Differenzialdiagnose für immunologisch bedingte Eigennierenerkrankungen dar. Diese sollten mittels einer Nierenbiopsie abgeklärt werden.
- Eine antineutrophile Zytoplasmaantikörper (ANCA)-assoziierte Vaskulitis der Niere kann mit der Bildung von Halbmonden einhergehen. Basis der Halbmonde sind Nekrosen und diese Veränderungen stellen aufgrund des akut eintretenden Nierenversagens einen medizinischen Notfall dar.
- Neben serologischen und klinischen Parametern ist die Nierenbiopsie bei autoimmunologischen Systemerkrankungen das diagnostische Mittel der Wahl.
- Neue Therapieansätze ergeben sich auch Erkenntnissen der Rolle des Komplementsystems.
